# A lateral approach defect closure technique with deep fascia flap for valgus knee TKA

**DOI:** 10.1186/s13018-015-0316-3

**Published:** 2015-11-10

**Authors:** Jun Jiang, Julio C Fernandes

**Affiliations:** Arthritis Clinical & Research Center, Peking University People Hospital, #11 Xizhimen South Avenue, Xicheng District, Beijing, 100044 China; Orthopaedic department, Hopital Du Sacre-Coeur De Montreal, 5400, Boul. Gouin Ouest, Montreal, Quebec H4JIC5 Canada

**Keywords:** Deep fascia flap, Capsular defect, Valgus knee, TKA, Lateral parapatellar approach

## Abstract

**Background:**

Routinely, we use a midline skin incision and lateral parapatellar approach of the knee to perform valgus knee TKA (total knee arthroplasty). It is generally very difficult to close the lateral capsular defect after valgus knee TKA, especially for severe valgus and flexion knee deformity.

**Methods:**

We describe a new surgical technique to close the lateral capsular defect with a deep fascia flap. From 2009 to 2012, we used the new technique to close lateral capsular defects for nine valgus TKA in eight patients. The wound healing, infection, range of motion, and postoperative X-ray Laurien view were evaluated.

**Results:**

According to follow-up, we found that this technique can reduce the risk of intra- and postoperative complications (exposure of knee prosthesis, larger subcutaneous hematoma, poor wound healing, and higher risk of infection) and improve clinical outcome of total knee replacement (good range of motion and patellar tracking). There is no need for lateral parapatellar capsule Z-plasty during incision or filling the distal capsular defect with fat pad or composite meniscal-capsular-fat pad.

**Conclusion:**

Closing lateral capsular defect with a deep fascia flap for valgus knee TKA through a lateral parapatellar approach is a new and effective surgical technique.

## Introduction

In total knee arthroplasty (TKA) for valgus knee, two approaches may be performed: medial or lateral parapatellar incisions. However, it may be more difficult to reach the posterolateral corner for the releases using a medial parapatellar approach in arthritic knees with valgus deformity than in those with varus deformity, especially for moderate (valgus angle >15°, but <30°) and severe (>30°) valgus kneea associated to flexum deformities. On the contrary, using the lateral approach, the lateral structures visualization is better, and lateral release can be performed directly during the approach and not as an accessory step. Some authors reported that a better clinical outcome is obtained through a lateral parapatellar approach than through a medial one when performing a TKA for a valgus knee [1].

A lateral capsular defect is created after TKA through a lateral parapatellar approach for valgus knee, which usually cannot be sutured directly because of high tissue tension. Fiddan et al. suggested that no attempt should be made to directly close the lateral defect completely [1]. This defect at the distal end or in the middle of the lateral capsular incision causes prosthetic exposure and joint seal problems, larger subcutaneous hematomas, difficult wound healing, and higher risk of infection. Keblish, Buechel, and Bassaine et al. reported the distal capsular defect can be repaired with a fat pad flap or a composite meniscal-capsular-fat pad flap [2–4]. This fat pad is fragile and can be easily torn during knee flexion, especially with large defect. Therefore, we report a new, simple, and effective surgical technique for lateral capsular defect closure with a deep fascia flap in valgus knee TKA operated through a lateral parapatellar approach.

## Materials and methods

Our study was approved by the clinical research ethical committee of Hopital du Sacre-Coeur De Montreal, Canada.

### Patients demographics

From 2009 to 2012, we performed nine valgus knee TKA surgeries through a lateral parapatellar approach in eight patients. There was one male patient and seven female patients with the average 66.6 years old (57–76 years). According to the knee valgus angle (femoral-tibial angle), there were four mild valgus knees, three moderate valgus knees, and two severe valgus knees. There were three rheumatoid arthritis valgus knees and six osteoarthritis valgus knees. The valgus deformity could be reduced in four mild valgus knees but could not be reduced (fixed valgus knee deformity) in three moderate valgus knees and two severe valgus knees.

### Surgical procedure

**Fig. 1:**
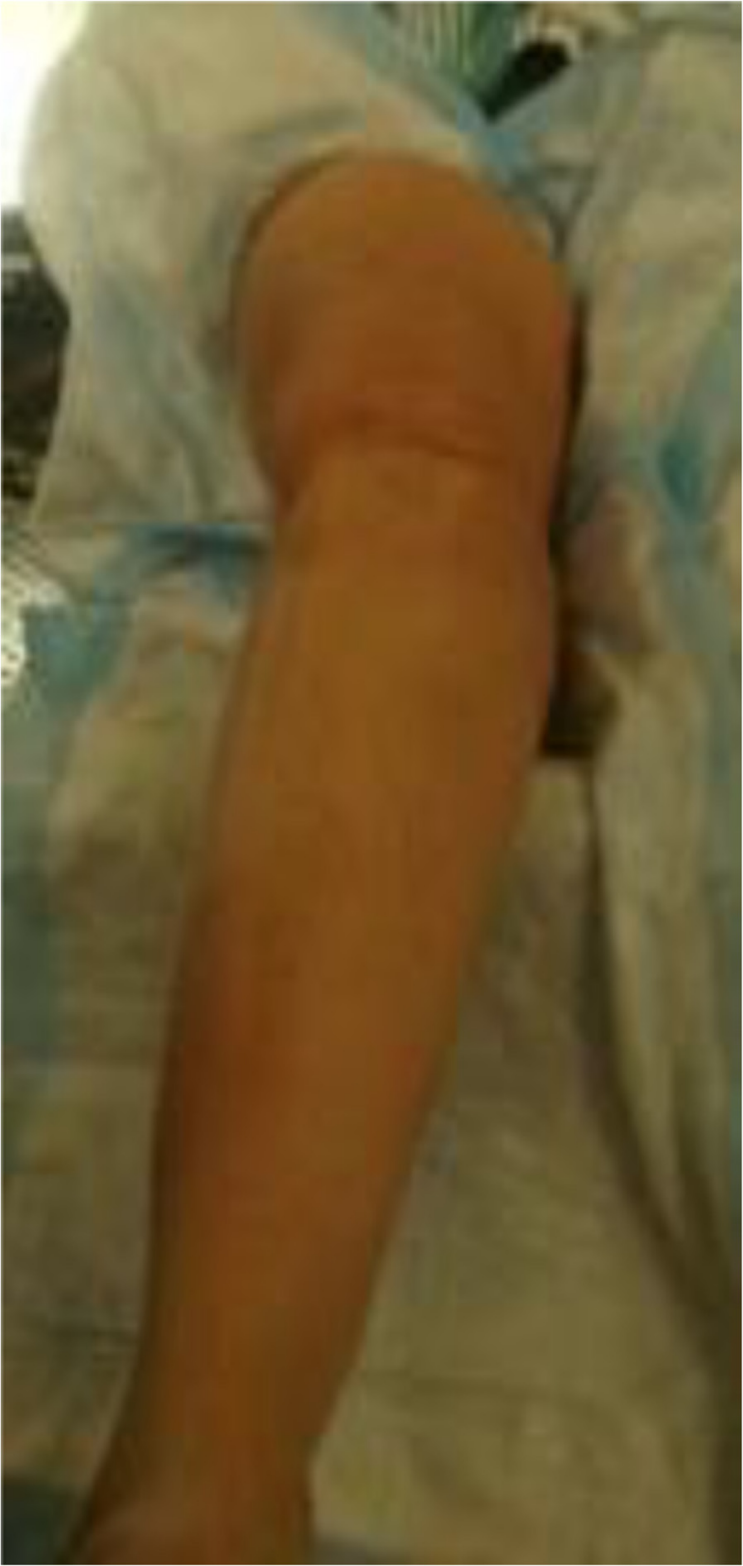
Right knee with moderate valgus knee deformity (valgus degree is about 25°)

A midline skin incision of the knee was performed instead of a lateral one in order not to interfere with the medial dislocation of the patella and preserve a midline access for future revision surgeries, whenever necessary. The staggeredness between midline skin incision and lateral parapatellar capsular approach is also good for preventing infection (Fig. 1).

**Fig. 2:**
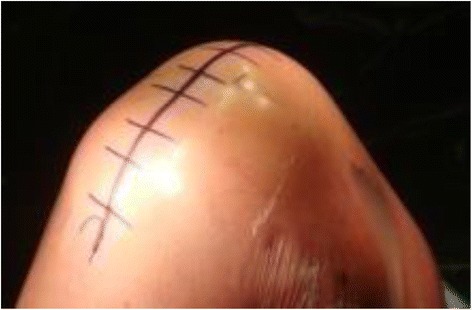
The midline skin incision of right knee

**Fig. 3:**
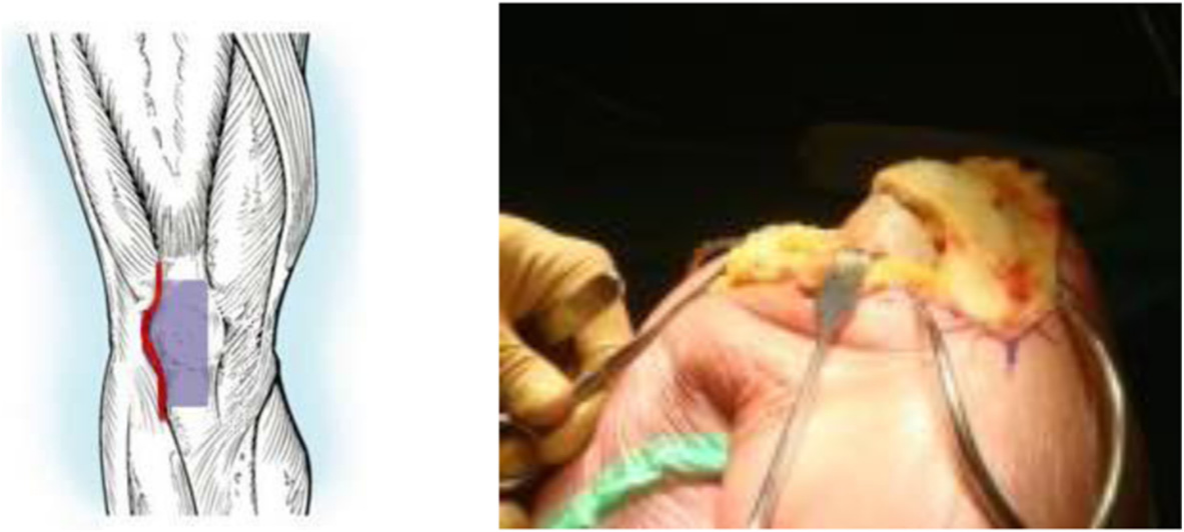
The lateral parapatellar capsular approach

Lateral parapatellar approach is performed as follows. The quadriceps tendon is clearly defined and incised longitudinally in the midline, starting 5 cm above the superior pole of the patella then curving along the lateral border of the patella down to the lateral side of tibial tubercle (Fig. 2). Any adhesions in suprapatellar pouch and medial/lateral gutters are excised. The incision in the quadriceps tendon is extended proximally beneath the subcutaneous fat tissue and skin for 2 to 3 cm with scissors in order to release the contracted vastus lateralis completely from the patella and quadriceps tendon. The patella is then displaced medially without eversion with a thin Homann retractor to keep it away from the medial femoral condyle, which gives exposure for the femoral bone resection (Fig 3). With lateral parapatellar approach, the valgus knee deformity would be corrected through release of lateral contracted soft tissue with the following sequence: (1) pie-crusting of iliotibial tract above the joint line which should be performed before incision of lateral capsular approach, (2) subperiosteal release of iliotibial tract insertion from Gerdy tubercle, (3) release of the posterolateral capsule around lateral tibial plateau, (4) subperiosteal release of lateral collateral ligament and popliteal tendon from the lateral femoral condyle working proximally, and (5) release of the lateral head of gastronemius muscle and the biceps tendon [4].

The aforementioned soft tissue balance should be performed in three stages. The first balance stage should be pie-crusting of iliotibial tract above joint line, elevating the iliotibial tract subperiosteally from Gerdy tubercle, and release of posterolateral capsule around lateral tibial plateau. If there is a correctable valgus deformity after first balance stage, the subperiosteal release of the posterolateral capsule should not continue to the anterior half of the lateral tibial plateau. The next two balance stages should not be performed until the knee prosthesis trial test is completed [2]. After the knee prosthesis trial, soft tissue balance should be checked again. If there is still valgus deformity especially in knee flexion, soft tissue balance should proceed to stage II as described by Buechel, including subperiosteal release of popliteal tendon and lateral collateral ligament from the lateral femoral condyle [3]. After sequential release of these tissues, it is very important to keep checking the balance both in knee extension and flexion. A stage III release, including release of lateral head of gastrocnemius muscle and biceps tendon release, has been described by Buchel, but has never been performed during our valgus knee TKA surgery.

The rotational alignment of the tibial plateau prosthesis should be determined according to the tibial prosthesis trial position in knee extension [1], because knee stability in extension is very important during walking on level. If the patella is to be resurfaced, it should be performed with the knee in extension because the patella will be everted easily. During cementing of the femoral and tibial components, the patella should be displaced medially to give adequate exposure.

After knee prosthesis replacement, the lateral capsular incision should be sutured with the knee in 30° flexion. The lateral cuff of the quadriceps tendon with proximal vastus lateralis should be sutured to the central quadriceps tendon under gentle tension, to effectively reposition the vastus lateralis proximally. The distal portion of lateral capsular incision is also sutured until the tension is too high to be sutured directly. So, there is often a defect in the midportion of the lateral parapatellar capsular incision.

**Fig. 4:**
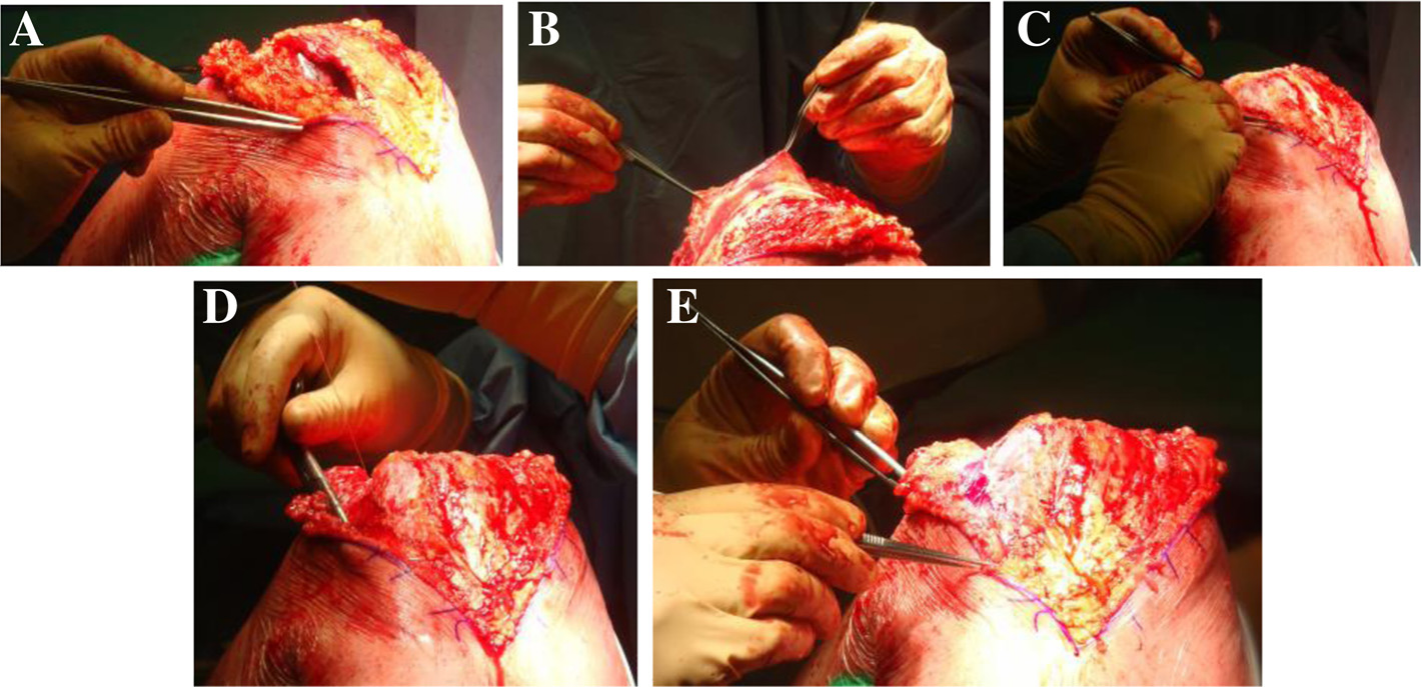
**a** After TKA of valgus knee with lateral parapatellar approach, a lateral capsular defect in the midportion will be seen after proximal and distal capsular closure. **b** A deep fascia from the anterior patella and quadriceps tendon is dissected to form a flap. **c** The deep fascia flap is reversed for covering the lateral capsular defect. **d** Suturing the lateral capsule with 2–0 running absorbable suture. **e** The covering of the lateral capsular defect is examined

Here, we introduce a new surgical technique to address the midportion defect of the lateral parapatellar capsular incision. According to the defect size at the midportion of the lateral parapatellar capsular incision, a deep fascia flap with about 2 mm thickness is dissected over the patella/quadriceps tendon/patellar tendon. Then, the deep fascia flap is flipped laterally with pedicle attachment preserved at the fascia flap base to cover the lateral capsular incision defect. The flipped deep fascia flap is sutured to the lateral capsule using 2–0 running absorbable suture. Then, the deep fascia flap pedicle is also sutured at the flap base for reinforcing to prevent rupture during postoperative knee flexion. After completing the suture, the lateral capsular defect should be covered with this deep fascia flap. The anti-tension capacity of the deep fascia flap is measured through full knee extension to 120° knee flexion, and should show good anti-tension capacity during full flexion (Fig. 4).

#### Clinical data

In 2 weeks postoperatively, subcutaneous hematoma and wound healing were evaluated. Acute (infection during 6 months postoperatively), delayed (infection during 6~24 months postoperatively), and late infection (infection after 24 months postoperatively) were also evaluated. The valgus angle was measured in weight bearing X-ray Ap view, and lateral patellar tilt angle was measured in Laurin axial X-ray view, and compare them between preoperatively and postoperatively. Valgus angle in Ap view is defined as the intersection angle between anatomical axis of femoral bone and tibial bone; the lateral patellar tilt angle in axial view is defined as the intersection angle between the patellar transverse axis and the line through the highest point of lateral and medial femoral condyles. The knee range of motion was also assessed.

## Results

The mean follow-up period is 55.8 months. There was no subcutaneous hematoma formation or wound healing problems at 2 weeks postoperatively. There was also no acute, delayed, and late infection. The preoperative valgus angle ranged  from 12° to 31° (with mean value 16.13°); the postoperative valgus angle is from 2° to 7° (with mean value 4.75°), which is significantly lower than preoperatively (*P* < 0.01). The preoperative lateral patellar tilt angle ranged from 0.19° to 13.8° (with mean value 3.22°); the postoperative lateral patellar tilt angle averaged from 0.81° to 23.7° (with mean value 10.73°) which is significantly higher than preoperatively (*P* < 0.01). The knee range of motion is between 120° flexion to 0° extension.

## Discussion

Compared with the medial parapatellar approach, the lateral parapatellar approach for TKA of moderate to severe valgus knee has the following advantages: (1) directly release lateral soft tissue contracture during lateral approach, (2) decreases subcutaneous undermining for lateral iliotibial tract pie-crusting, (3) improved access to the posterolateral corner with the internally rotating tibia, (4) allows for better sequential release based on flexion/extension gap balance, (5) centralizes the deep quadriceps tendon which optimizes patellar tracking, (6) preserves more vascularity because medial structure is untouched, and (7) postoperative rehabilitation is not delayed because vastus medialis remains intact [6].

But, in TKA for moderate to severe valgus knee deformity with lateral approach, prosthetic covering, and joint sealing after valgus knee TKA may be a problem. The lateral capsular incision cannot be sutured directly because of large capsular defects and high tension. Increased tension and lack of a soft tissue layer between skin and prosthesis can lead to larger subcutaneous hematoma, wound healing problem, potential implant exposure and even infection—a potentially devastating complication of TKA. How to address the problem?

Lateral capsular Z-plasty (lateral capsule oblique incision in coronal plane) proposed by Czech orthopedic surgeon Stehlik et al. is one method to avoid the problem [5]. The lateral capsule is incised obliquely (from superficial laterally to deep medially) at about 2 cm lateral to the patella, extending up to 4 cm from laterally to medially. By transiting superficial-medial layer medially and deep-lateral layer laterally, and suturing these two layers in the abutting border, so medial transposition of the patella and lateral capsular defect closure after valgus knee TKA are achieved [7]. But, there are two disadvantages for capsular Z-plasty: (1) Dissection between skin and capsule may be needed for capsular Z-plasty, which can cause much subcutaneous undermining with risk of skin necrosis. (2) For severe valgus deformity, there will be a distal capsular defect which needs fat pad or composite meniscal-capsular-fat pad flap to cover.

Keblish, Buechel, and Bassaine et al. also reported the distal capsular defect can be covered with Hoffa’s fat pad flap or composite meniscal-capsular-fat pad flap flipped laterally with medial pedicle preserved in patellar tendon [2–4]. But, the fat pad is not strong enough and can be easily torn by knee flexion during postoperative rehabilitation, especially for large defect. It will cause subcutaneous hematoma and fat liquefaction which will result in poor wound healing and higher risk of infection.

Classical lateral approach for valgus knee usually needs a lateral skin incision [2, 6] which may present problems when a TKA revision is needed later. And, because of no staggeredness between lateral skin incision and fascia/capsular incision, there is a higher risk of infection for TKA. Lateral skin incision will result in larger medial skin flap, which may have problem of skin necrosis because of poor vascularity. Using our new technique, a standard midline skin incision can be performed which will avoid the disadvantages mentioned above.

The supporting structures underneath skin and fat tissue on the lateral side of the knee have been described as consisting of three layers. Layer 1 contains the deep fascia, the iliotibial tract, and the biceps femoris with its expansion posteriorly. Layer 2 is formed by the lateral patellar retinaculum. Layer 3 is composed of the lateral capsule [7].

The deep fascia is more tensile than fat tissue which can bear tension caused by knee flexion during postoperative rehabilitation. So, there is low risk for subcutaneous hematoma, fat liquefaction, and knee joint infection because of good joint seal and low prosthesis exposure risk. To prevent rupture of medial pedicle of fascia flap, we used 2–0 absorbable running suture to reinforce its strength.

Another advantage of deep fascia flap for covering lateral capsular defect is keeping its laxity after the release of lateral contracted structure, which is useful for patellar tracking. Surprisingly, in our follow-up result, the postoperative lateral patellar tilt angle is more than preoperatively, even though the patella was well alligned on the femoral throclea. That means there could still have excessive lateral tension even after covering lateral capsular defect with deep fascia flap. Moreover, in valgus knee TKA, there is a higher risk to place the femoral implant in excessive  internal rotation because of lateral femoral dysplasia, which can cause larger postoperative patellar tilt angle.

## Conclusion

Closing the lateral capsular defect with a deep fascia flap for valgus knee TKA exposed through a lateral parapatellar approach is a new and effective surgical technique. This technique is easy to be performed after valgus knee TKA with enough strength and tension compared to fat pad and composite meniscal-capsular-fat pad flap.
